# Sequential Automated Analysis System for Lower Oxygenated Organic Compounds in Ambient Air

**DOI:** 10.6028/jres.093.053

**Published:** 1988-06-01

**Authors:** Ikuo Watanabe

**Affiliations:** The Institute of Public Health, 4–6–1 Shirokanedai, Minato-ku, Tokyo 108, Japan

A completely automated system controlled by a microcomputer was developed for hourly analyses of lower oxygenated organic compounds (LO*_x_*) in ambient air at the sub-ppb level. This system has some advantages, compared with manual procedures, including 1) good repeatability, 2) easy data processing, 3) easy accumulation of extensive data throughout the day and night, and 4) reduction of labor. Consecutive measurements using this system for 6–15 days have been carried out several times since November 1985 in Tokyo.

## 1. Introduction

LO*_x_* are formed by the degradation of atmospheric hydrocarbons by free radicals, and are also emitted from various sources [1]. These compounds have been identified and measured in air [1–4], however, little is known about their concentration in the environment.

The accumulation of a large amount of data is essential for elucidation of the phenomena induced by the existence of LO*_x_*. For this purpose, a practical and durable automated system was developed.

## 2. Experimental

### 2.1 Sampling Trap

Four kinds of adsorbents (Porapak–N, –Q, –T and Chromosorb 104) were examined for collection ability, and for desorption efficiency of LO*_x_* and their parent compounds by heating. The results showed Chromosorb 104 (C104) to be superior to the other 3 adsorbents when a stainless steel tube (3 mm i.d. ×16 cm) packed with 0.4 g of C104 was used for the sampling tube. The collection volume of this trap for LO*_x_* was estimated to be 2.4 L or more at 0 °C.

### 2.2 Sampling Air

An air sample was drawn at a height of 20 m from the ground and 3 m from the walls through a 20 m Teflon tube (6 mm o.d.) covered with a black tube. To reduce the loss of the compounds through the tube, the air sample was continuously drawn with a pump at a flow rate of 1 L/min. A portion of the air was sucked from the end of the main air stream for 30 min with a flow-controlled pump, through the sampling trap (kept at ca. 0 °C) using a flow rate of 45–50 mL/min to collect the LO*_x_* in the air sample.

### 2.3 Chromatographic System

**Figure 1:**
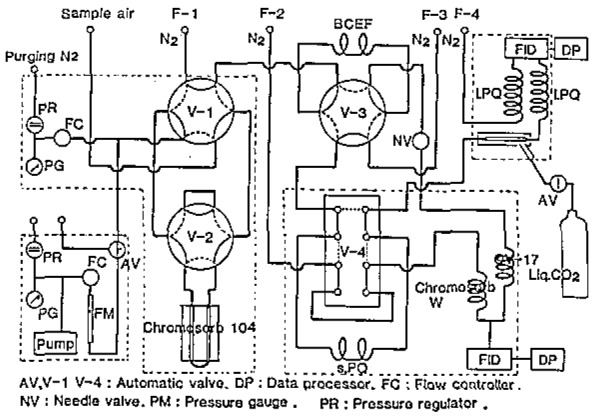
Schematic for automated sampling and analysis.

The system with an automated sampler is shown in figure 1. Two precolumns (BCEF and s.PQ in table 1) are used for the pre-fractionation of aliphatic hydrocarbons (AlHC) and aromatic hydrocarbons (ArHC), because some of AlHC overlap LO*_x_* in chromatograms and ArHC prolong the analytical time.

The peaks in chromatograms of air samples were periodically identified by GC/MS. If the method requires sample sizes of more than 10 L, another sampling method was used [i.e., a trap (3 mm i.d. ×18 cm Teflon tube) packed with quartz wool impregnated with water was installed between 1.PQ and FID, with the LO*_x_* peaks confirmed by their disappearance from the chromatograms].

The switching of the valves, fan, heater, cooler, GC, and data processor are controlled with a microcomputer and an interface. An air sample was ataken every hour for 30 min, and the chromatograms and the processed results were printed on chart paper and stored on floppy disks.

## 3. Results and Discussion

The reproducibility tests using a specially prepared gas containing LO*_x_* at ppb levels showed that the overall system precision for LO*_x_* was 1–5% (RSD) during one day, and the changes of the retention times were less than 0.5% (RSD). Detection limits with signal-to-noise of 4 were 0.5 ng for methanol, 0.1 ng for acetaldehyde and 0.1–0.5 ng for others. This corresponds to ca. 0.3 ppb of methanol and ca. 0.04 ppb of acetaldehyde respectively, in a 1.5 L air sample.

**Figure 2:**
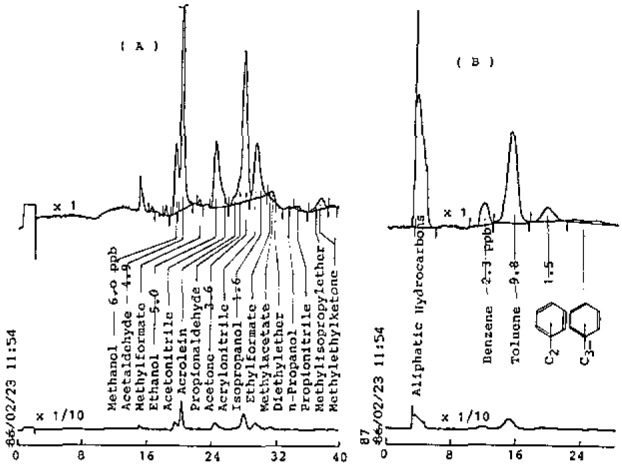
Gas chromatograms of ambient air (1.38 L).

A typical chromatogram obtained with this system is illustrated in figure 2, showing the concentrations of some LO*_x_* and ArHC species. Since the acetone and isopropanol peaks might contain other compounds, their values shown in figure 2 are considered to be tentative.

The monitoring of LO*_x_* for 6–15 days has been carried out several times in the metropolitan area of Tokyo since November 1985, and data on more than 1800 samples have been obtained. Acetaldehyde, C_1_–C_3_ alcohols, and acetone were constantly observed in chromatograms. Acrolein, C_2_–C_3_ esters, propionaldehyde, C_4_–C_5_ ethers, and methylethylketone were occasionally detected. This system has worked satisfactorily for more than 2500 hours without any exchange of parts in the devices, and has proved to be practical and durable.

## Figures and Tables

**Table 1:** Operating conditions for gas chromatograph

	Packings	Tube (SUS)	GC conditions
P1	N,N-bis(2-cyanoethyl) formamide [BCEF] on Chromosorb W (60/80)	3 mm (i.d.) 1.5 m (long)	Room temperature (18–30 °C) N_2_ (38 mL/min)
P2	Porapak Q (50/80) [s.PQ]	2 mm (i.d.) 25 cm (long)	124 °C(12.5 min) –4 °C/min(15 min) –184 °C(25 min) N_2_ (41 mL/min)
AC	Porapak Q (80/100) [1.PQ]	1.5 mm (i.d.) 4 m (long)	38.25 °C–1.5 °C/min(14.5 min)–20 °C/min(2.5 min)–10 °C/min(3 min)–2 °C/min(20 min)–180 °C(13 min) N_2_ (33 mL/min)
